# Scalable and Robust Fabrication, Operation, and Control of Compliant Modular Robots

**DOI:** 10.3389/frobt.2020.00044

**Published:** 2020-04-07

**Authors:** Nialah Jenae Wilson, Steven Ceron, Logan Horowitz, Kirstin Petersen

**Affiliations:** ^1^Collective Embodied Intelligence Lab, Mechanical and Aerospace Engineering, Cornell University, Ithaca, NY, United States; ^2^Collective Embodied Intelligence Lab, Electrical and Computer Engineering, Cornell University, Ithaca, NY, United States

**Keywords:** self-reconfigurable, modular robots, soft robots, robot kinematics, simulation environment, path planning

## Abstract

A major goal of autonomous robot collectives is to robustly perform complex tasks in unstructured environments by leveraging hardware redundancy and the emergent ability to adapt to perturbations. In such collectives, large numbers is a major contributor to system-level robustness. Designing robot collectives, however, requires more than isolated development of hardware and software that supports large scales. Rather, to support scalability, we must also incorporate robust constituents and weigh interrelated design choices that span fabrication, operation, and control with an explicit focus on achieving system-level robustness. Following this philosophy, we present the first iteration of a new framework toward a scalable and robust, planar, modular robot collective capable of gradient tracking in cluttered environments. To support co-design, our framework consists of hardware, low-level motion primitives, and control algorithms validated through a kinematic simulation environment. We discuss how modules made primarily of flexible printed circuit boards enable inexpensive, rapid, low-precision manufacturing; safe interactions between modules and their environment; and large-scale lattice structures beyond what manufacturing tolerances allow using rigid parts. To support redundancy, our proposed modules have on-board processing, sensing, and communication. To lower wear and consequently maintenance, modules have no internally moving parts, and instead move collaboratively via switchable magnets on their perimeter. These magnets can be in any of three states enabling a large range of module configurations and motion primitives, in turn supporting higher system adaptability. We introduce and compare several controllers that can plan in the collective's configuration space without restricting motion to a discrete occupancy grid as has been done in many past planners. We show how we can incentively redundant connections to prevent single-module failures from causing collective-wide failure, explore bad configurations which impede progress as a result of the motion constraints, and discuss an alternative “naive” planner with improved performance in both clutter-free and cluttered environments. This dedicated focus on system-level robustness over all parts of a complete design cycle, advances the state-of-the-art robots capable of long-term exploration.

## 1. Introduction

Modular self-reconfigurable robots are composed of active modules capable of rearranging their connection topology to adapt to dynamic environments, changing task settings, and partial failures (Yim et al., 2007). It is desirable to increase the number of modules to increase the potential for adaptability and redundancy, however, scaling up the collective size poses several challenges (Brunete et al., 2017). Controllers must be capable of efficiently exploring the configuration space and providing introspection to cope with internal and external changes. The module hardware must be inexpensive and fast to produce, work reliably, and require little maintenance. Consequently, isolated efforts to develop scalable control and hardware do not necessarily result in system-level robustness. Rather, to facilitate large numbers of robots in the first place, we argue for the importance of incorporating robustness into all levels of design, and demonstrate how this approach leads to tightly co-dependent parameters across hardware and software. In this paper, we discuss our design approach, an early hardware prototype, and custom controllers. Our focus is explicitly on enabling long-term robustness of an autonomous, self-reconfigurable, modular robot through a hardware-software design cycle, with the idea that we can build on such a robust platform in the future to achieve more advanced behaviors.

**Figure 1:**
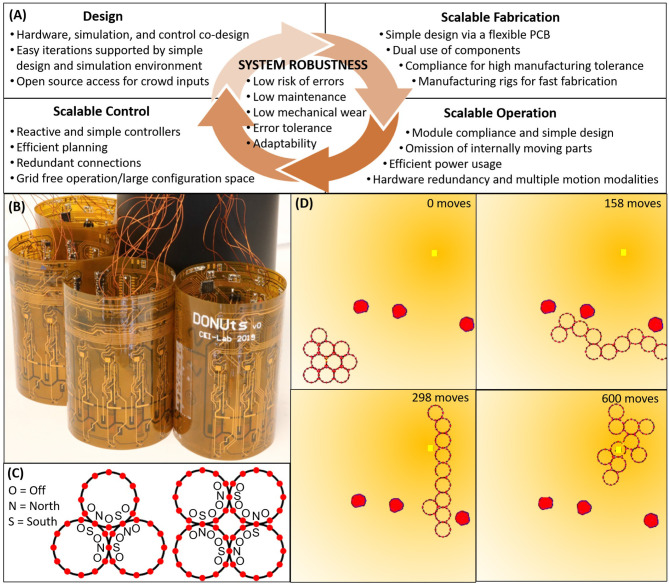
**(A)** Interdependent design guidelines centrally motivated by system level robustness. **(B)** Simulated DONUts moving along an environmental gradient, where the yellow square denotes the origin of the gradient, and the red polygons obstacles. **(C)** DONUt modules with onboard computation, sensing, and switchable magnets to facilitate collective communication and motion. **(D)** Magnets which can be switched to either polarity and off to permit a large range of configurations.

Figure 1A provides an overview of the measures we have taken to ensure system-level robustness, and how many of these design decisions carry over between fabrication, operation, and control. Related to the *design* itself, system robustness is mediated by (1) the simultaneous development of hardware and software; (2) ease of iterations, e.g., through realistic simulation environments that let the designer focus on high level behaviors, as well as simple hardware that supports easy extensions; and (3) open access to permit a wide range of users and inputs. To support inexpensive, fast, and therefore *scalable fabrication* we focus on (1) simple designs with minimal components; (2) mechanical compliance to permit higher manufacturing tolerances; and (3) manufacturing rigs to support non-expert labor. These design parameters correlate with those of *scalable operation*, e.g., because (1) compliance lets modules interact safely with each other and with external objects; (2) compliance permits large scale connectivity despite poor manufacturing tolerances; and (3) hardware simplicity limits the risk of failure. Other operation-specific considerations include the ability of modules to operate, sense, and perceive independently from others; the ability to stay connected without continuous use of power; the ability of modules to move in a multitude of ways to overcome partial failures; and the potential to lower mechanical wear by omitting internally moving parts. All of these design choices warrant custom *controllers* and to support system robustness, we focus on (1) reactive (over deterministic) behaviors that could adapt to dynamic perturbations; (2) naive and simple control schemes that scale well with the number of robots; (3) minimum energy expenditure through efficient path planners; (4) connection redundancy to avoid single module failures from causing complete collective failure; and (5) enabling a large configuration space that facilitates system adaptability to unforeseen perturbations.

More specifically, we introduce a novel planar, modular robot composed of compliant modules moving in unison. We refer to the robotic modules as “DONUts” (Deformable Self-Organizing Nomadic Units) for their visual kinship (Figures 1B–D). To support simple and fast manufacturing, DONUt modules are composed of a single flexible printed circuit board (PCB) wrapped in a loop and populated with sensors, actuators, processors, and room for batteries. To mitigate wear, the DONUts have no moving parts; rather, they move as a collective by activating and deactivating Simplified Electro-Permanent Magnets (SEPs) on their perimeter. These magnets can be polarized in either direction or turned off to enable a very large configuration space and consequently collective adaptability (Figure 1C). Furthermore, they do not require continued application of power to maintain polarization which saves energy. To lower fabrication cost and risk of errors, we minimize the number of components, e.g., by making double use of the PCB as a chassis and the SEPs for communication. The passive compliance introduced with the flexible PCB permits large lattice configurations despite rapid, imprecise manufacturing. The compliance and low driving voltages also enable the modules to interact safely with each other and with surrounding objects.

We further develop DONUt-specific coordination schemes, low-level primitives for module operation, as well as an open source simulation environment to support controller development. We refrain from imposing artificial constraints on module motion beyond what the hardware is capable of. This means that the modules operate in a grid-free environment and can achieve a much larger set of connection topologies to adapt to the task at hand. Toward real-world operation, we furthermore focus on reactive configurations, rather than predetermined shape transitions as is common for modular robots. Specifically, in a simulated energy harvesting scenario, we investigate how such modules may perform gradient tracking toward a light source in clutter-free and cluttered environments (Figure 1D). We choose this specific task, because it supports reactive and scalable behavior and because it highlights the benefits of grid-free operation. To evaluate the performance, in terms of path efficiency, of our controllers, we compare them against paths generated by an all-knowing Oracle planner in clutter-free environments. We explore a locally optimal A* search-based controller and how we may incentively redundant connection topologies for more error tolerant operation. We compare this to a “naive” iterative control scheme that scales better with the number of modules in both computation and memory, finding comparable performance. We further discuss particular connection topologies which may impede progress due to the hardware-specific motion constraints, and show how these may be circumvented using the naive controller. We also allude to how energy expenditure is used across modules in the collective, which is an interesting area for future work. In this paper, we focus only on centralized coordination, however, all of the methods may be adapted for decentralized coordination at the expense of communication.

Although more work is needed to demonstrate full-scale practical collective operation, the work in this paper illustrates the highly interdependent design choices that lay the foundation for a scalable and robust modular robot. The following sections detail (1) related work of both controllers and hardware; (2) a hardware prototype composed of compliant modules with individual computation, communication, sensing, and collective motion; (3) an inexpensive, quick manufacturing process for both modules and components, based on pre-populated, flexible PCB and a rapid SEP winding mechanism; (4) a characterization of module deformation, mobility, sensing, and communication; (5) an open source kinematic simulation framework for the DONUts informed by low-level motion primitives and experimentally obtained sensor performance characteristics; and (6) a comparative study of two controllers for efficient and error tolerant gradient tracking with the DONUts in environments without an occupancy grid.

## 2. Related Work

The framework described in this paper combines and builds on findings from many sources spanning both hardware and coordination. In the following sections, we describe these in turn.

### 2.1. Modular Robot Platforms

Past research on hardware for modular self-reconfigurable robots includes design of inexpensive and durable mechanisms for actuation, docking, communication, and power distribution (Brunete et al., 2017). Low maintenance requirements are especially important for this class of robots, as they scale linearly with the number of modules required. Module cost and fabrication time are equally important factors, but are somewhat mitigated by the fact that unit price decreases significantly with mass fabrication. Additionally, the module weight and stiffness determines both structural stability and how many modules can be moved at once.

The majority of modular robots consist of rigid components assembled into either a fixed form factor (Jorgensen et al., 2004; Goldstein et al., 2005; Daudelin et al., 2018; Zhu and El Baz, 2019), or into modules which can actively deform to produce motion (Rus and Vona, 2001; Ishiguro et al., 2006; Karagozler et al., 2007; Li et al., 2019). Recently, merging with soft robotics, pneumatically-driven modules with infinite degrees of (passive) freedom have also been shown (Lee et al., 2016; Vergara et al., 2017). These have the benefit of overcoming small manufacturing defects that otherwise scale poorly in large lattice-structures. The most successful demonstrations of these robots currently rely on traditional electro-mechanical actuators for reconfiguration, such as DC motors (Daudelin et al., 2018). However, researchers are also exploring designs that require fewer components and (1) have no internal moving parts which are prone to wear (Goldstein et al., 2005; Vergara et al., 2017; Zhu and El Baz, 2019), (2) rely solely on collective motion over individual module mobility (Goldstein et al., 2005; Li et al., 2019), and (3) exploit non-mechanical latches, such as switchable magnets (Goldstein et al., 2005; Gilpin and Rus, 2010; Zhu and El Baz, 2019), electrostatics (Karagozler et al., 2007), and meltable plastic and alloys (Neubert et al., 2014; Swissler and Rubenstein, 2018). Note that the last two options are superior for connection strength, but require high voltage generation or power usage, respectively. The DONUts are intended for rapid reconfiguration, standardize operation, and will not experience high tensile force, therefore we base our design on switchable magnets.

Currently, the closest “relatives” of the DONUts are the Caroms (Goldstein et al., 2005) and the Nonoperable (Gilpin and Rus, 2010), both planar modular robots. In the former, round, rigid modules can move in six discrete steps around each other using switchable magnets. This is still an active research platform, especially in terms of controllers, power, connectors, and communication (Campbell et al., 2005; Kirby et al., 2007; Naz et al., 2018; Piranda and Bourgeois, 2018). The DONUts rely on a similar means of locomotion, but are compliant, simpler to manufacture, and have the potential to be teacherless. The Nonoperable are small form-factor cubes with switchable magnets used both for inter-module docking, power transfer, and communication; module movement comes from external forces. They involve a quick manufacturing procedure by wrapping a flexible PCB around a rigid frame, enabling deflections to overcome manufacturing defects.

It is worth noting our specific choice of an SEP docking mechanism. In the Caroms and Nonoperable, the switchable magnets were electromagnets and electro-permanent magnets, respectively. The former has high power consumption when on, and the latter can only be switched off or on in one polarity. To limit power consumption and to permit a wider range of configurations (Figure 1C), we instead leverage SEPs (Zhu and El Baz, 2019) which can switch polarities and be turned off. We further explore different SEP designs to lower module weight and enable stand-alone operation.

In summary, the design of the DONUts combines many of these past findings, including: (1) passive module compliance to overcome manufacturing defects, (2) collective motion via switchable magnets to decrease mechanical wear, and (3) a very simple fabrication process to improve system scalability.

### 2.2. Coordination of Modular Robots

Path planners for modular, lattice-based robots typically focus on shape transition, i.e., how to plan admissible and energy efficient paths for all modules from one configuration to another (Pamecha et al., 1997; Walter et al., 2005). Past literature on reactive reconfiguration to reach a goal in a cluttered environment is much more sparse, but has been shown with slime mold-inspired, crystalline, and prismatic modules (Kubica et al., 2001; Rus and Vona, 2001; Butler et al., 2004; Ishiguro et al., 2006; Li et al., 2019), through coupled oscillators, traditional path planners, and cellular automata, respectively. All of these were based on distributed controllers and hardware with active degrees of deformation to help the modules move. In contrast, the DONUt modules briefly presented in Ceron et al. (2019a), have only passive compliance. Although this passive compliance is not currently part of our simulation framework, the presented control algorithms are only dependent on the connection topology and sensed objects, not the actual robot morphology, and could therefore work on the real hardware. We reason further about the benefits of module deformability and strain sensing in Ceron et al. (2019b).

The majority of research has focused on distributed controllers, e.g., through agent automata and globally imposed, or module-generated, gradients (Butler et al., 2004; Stoy and Nagpal, 2004). Centralized path planners for optimal shape transition become computationally intractable as the number of modules grow. This is typically overcome through careful preplanning (Walter et al., 2002; Daudelin et al., 2018) or sub-optimal planners dealing with hierarchical layers of modules (Bhat et al., 2006). The planning is further simplified through discrete occupancy grids (triangles, squares/cubes, and hexagons/rhombic dodecahedrons). Controllers for the Caroms, for example, typically discretion the world into hexagonal cells (Walter et al., 2005; Bhat et al., 2006). Although this approach is convenient mathematically, it also artificially limits the set of achievable configurations, which becomes especially critical in modules that are dependent on others to move.

Here, we explore centralized control schemes which adds no constraints on the module configuration beyond what the hardware is capable of. Similar to Ishiguro et al. (2006) and Li et al. (2019) we do not divide the world into a fixed occupancy grid, however, each module does have a finite set of connection points. Also, similar to many past controllers, path admissibility is ensured through a globally connected topology and consecutive movement of modules. Centralized controllers can suffer from a single point of failure and requires the need of a global sensor (or global communication), however, the algorithms we present rely on knowledge and plans which could be computed locally to overcome such weaknesses, at the expense of added synchronized communication.

## 3. Module Design and Characterization

**Figure 2:**
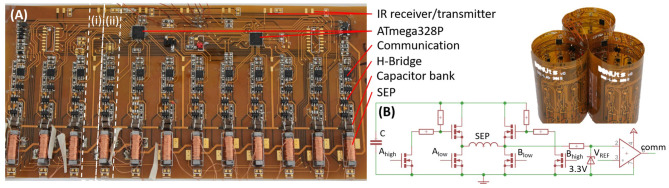
**(A)** A module PCB (unwrapped) and three modules (wrapped). The white dashed lines indicate (i) flexible and (ii) rigid regions due to the placement of components; IR sensors and amplifiers are not mounted in this photo, similarly, a few external wires appear only for initial debugging and powering purposes. **(B)** SEP driver and communication circuit.

We start by describing the SEPs, as they dominate the module infrastructure, power consumption, weight, and assembly time. We then detail the remainder of the hardware (Figure 2A), characterize the module ability to move, deform, communicate, and sense, and end with a discussion on scalability. As previously mentioned, our design considerations are based on enabling long term, stand-alone operation.

### 3.1. Docking Mechanism

**Figure 3:**
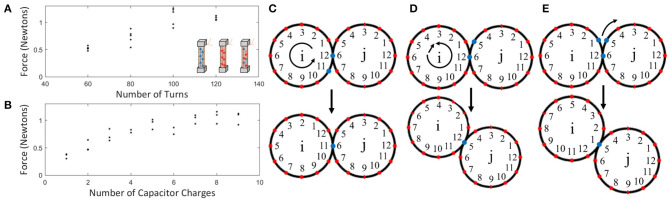
**(A)** SEP pull force when placed against a steel bar vs. the number of coil turns (five trials each). All measurements were taken by polarizing the SEP in one orientation with a strong magnet, then depositing a constant charge to flip the polarization. The insets show how the SEPs work conceptually, by changing the orientation of dipoles in the Alnico-material. **(B)** Pull force for an SEP with 100 turns, 40 AWG wire, and 4 min end caps. Each measurement was done by first orienting all dipoles in one direction (using 11 charges), and then depositing a number of charges in the opposite direction. **(C–E)** Conceptual movement by module *i* (red illustrates the position of the SEPs, blue those related to the move). **(C)** Rotating-motion, i.e., counterclockwise rotation of *i*. **(D)** Rotating-translating motion, i.e., counterclockwise rotation and translation by *i* to the adjacent SEP on *j*. **(E)** Gear-like motion, i.e., clockwise rotation by *i* along the perimeter of *j*.

SEPs consist of a low coercivity magnet wound with a copper coil and finished with ferrous end caps to induce and guide the magnetic field, respectively. By sending a high current pulse through the coil we can orient all the dipoles in the core to change its overall polarity; by applying a pulse of lower current magnitude, we can effectively turn off the magnet (Figure 3A inset). SEPs are advantageous for modular robots because (1) they have no internally moving parts which limit wear, (2) they remain polarized without continued supply of power which lowers maintenance, (3) they can be used for both movement and communication which minimizes the number of components, and (4) they can switch between opposite polarizations and off which supports a large range of module configurations. This section details our first SEP design and how it relates to the rest of the module design.

The SEP design considerations include part accessibility, the magnet geometry and coercivity, the geometry of the end cap, the number of turns in the coil, the wire gauge, and the amount of energy that can be transferred to the coil, which in turn is dependent on the supply voltage, series resistance, and pulse duration. These considerations come with trade-offs: a stronger SEP will facilitate better bonding strength and require fewer on-board SEPs needed for actuation, whereas a weaker SEP weighs less and can therefore work on a lower weight module that is easier to move. Our SEP design is based on a careful balance of these parameters. Small-scale, off-the-shelf, low coercivity magnets are rare and therefore the availability of these dominated our design. We decided on a magnet made of Alnico grade 5, with a length of 3/8” and a diameter of 1/8”, available from Magnet Kingdom. The end caps are made of steel and manually cut to the dimensions 4 × 4 × 1.5 mom.

A high energy pulse, and therefore a high supply voltage, is needed to flip the dipoles in the Alnico magnet. To keep the modules light weight, small, and mobile, we target a single cell Lithium Polymer on-board battery with a 3.7 V output. To activate the SEPs, we boost the battery voltage from 3.7 to 26 V, using an AP3012KTR boost converter with ~80% efficiency. To avoid damaging the battery, we first charge a capacitor bank *C* = 1*mF* slowly (over 75 ms), and then discharge rapidly from this bank into the coil. We choose ceramic capacitors to provide a low *R*_*ESR*_. There are four 22*uF* capacitors in parallel placed next to each SEP. Our circuit design is modular, such that the capacitor bank can be discharged into any combination of SEPs simultaneously depending on the actuation sequence desired (Figure 2B). The maximum charge, *Q*, that can be soured from the bank is given by: *Q* = *CV* = 27.5*mC*. We used this circuit to help us find the remaining parameters of the SEPs experimentally.

Knowing the magnet material and dimensions as well as the available power, we next focus on the coil. Specifically, the achievable SEP pull force is directly dependent on the amount of current we can push through the coil, which in turn is dependent on the number of coil turns (or inductance) and the resistance in the coil:

(1) $$\begin{array}{l} I=V/R_{ESR}\left(1-e^{-tR_{ESR}/L}\right) \end{array}$$

where *V* is the SEP supply voltage, *R*_*ESR*_ is the series resistance in the supply *R*_*C*_, plus that in the coil *R*_*L*_, *L* is the inductance of the coil, and *t* is the time since the charge started. Thicker, longer wires however produce diminishing returns due to (1) the maximum steady state magnetization strength of the Alnico rod, (2) the limited power available, and (3) the fact that the copper adds to the weight of the module which in turn increases the necessary pull force to produce motion.

Through a number of experiments to evaluate weight vs. magnet strength, we decided to settle for 40 AWE (American Wire Gauge) copper wire. Figure 3A shows how the number of turns with this wire affects the SEP pull force. *F*_*max*_ was measured between an SEP charged with the circuit described above and a steel bar, using a micro load cell rated 0–780 g from Fidgets Inc. As expected, *F*_*max*_ increases with an increasing number of turns, until *R*_*L*_ starts to limit *I*. With 100 turns, we found *F*_*max*_ = 1.11 ± 0.15*N*. We then measured how the pull force was affected by the number of times an SEP (40 AWE, 100 turns) was charged after being fully polarized in the opposite direction, shown in Figure 3B. We found that the SEP reaches maximum pull force after being charged approximately 5 times. These SEPs weigh 0.95 g, with the coil and end caps contributing 0.15 and 0.30 g, respectively. With 12 SEPs located around the perimeter of a 46 mom diameter module, the weight of a full module is around 20.9 g without a battery and 25.4 g with one; i.e., the 12 SEPs make up approximately 45% of the full module weight. Based on the experiments above, we use five consecutive capacitor bank charges to flip the polarity of a SEP and 1 to simply turn it off. As part of future work, we hope to perform a model-based analysis to find more optimal parameters, with the aim of increasing the SEP strength, while decreasing the total module weight.

### 3.2. Actuation

To move the modules, we make two assumptions: First, the moving module moves only itself. Second, the module it is moving around is connected to many other modules keeping it relatively stationary. To move on a neighboring module, the moving module has to first inform the other about its desired move and polarity. If the module is transitionary between two modules it needs to ask its neighbor to pass the message along to the following module. We target several types of motion including on-axis rotation, rotation-translation, and gear-like rotation, as shown in Figures 3C–E, respectively. We imagine that the latter two modes are useful for general motion, and that the former is of use if a particular module sensor is broken, or if the collective wants to take more measurements from slightly different angles. We anticipate that a combination of these motion abilities will support system-level robustness.

**Figure 4:**
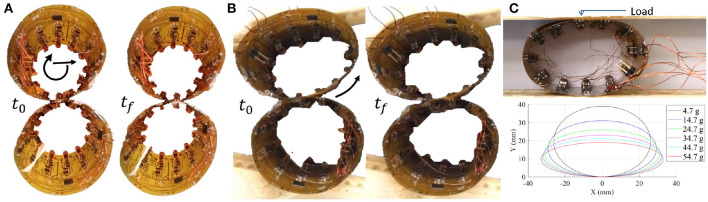
**(A)** Module moving about another module, with motions as described in Figure 3C. **(B)** Motion of compressed modules, with rotation similar to that described in Figure 3D. **(C)** Characterization of module deformation, conducted by placing weights on a module.

We found that rotation around the module axis is possible through the following sequence: (1) [S-O-N-N; N-S-S-N], (2) [S-O-O-N; N-S-S-N], (3) [S-N-O-N; N-S-S-N]; and (4) [S-N-S-N; N-S-S-N], where N, S, and O corresponds to north, south, and off, respectively. When enabling these types of motions we used a total of 11 capacitor bank charges to make sure that an SEP was polarized to the desired state. We further found that rotation-translation is feasible by conducting the following sequence of polarity switches: (1) [X-N-S-X; X-S-S-X], (2) [X-N-N-X; X-S-S-X], (3) [X-S-N-X; X-S-S-X], where X corresponds to any state. In step 1, the modules are connected between locations 2 in the array, and in step 3 between locations three in the array. This type of motion requires a total of 10 capacitor bank charges. We conducted a reliability test of the translation motion, and found that the module was able to successfully move 48 out of 50 times when no external forces were applied (Figure 4A). It should be noted that in these two experiments we used external power for experimental ease, but both tests were performed with the weight of a Li-Po battery on-board. It is also important to note that these moves require only one module to activate its SEPs. Therefore, although modules must first agree on the upcoming move with their neighbor, a movement does not require synchronous behavior.

Finally, we found that the current hardware only facilitates gear-like rotation in two scenarios: either when external compressive loads are applied as shown in Figure 4B, or when approximately half the weight is removed from the modules. More generally, we found that friction is an important factor in determining the motion that a module is capable of and that it is dominated by acceleration due to fast SEP switching. The acceleration, in turn, is determined by module inertia. According to Seiner's theorem, the inertia required for the module to move about a point on its perimeter is: *I* = *I_o_* + *mr*^2^, where *I*_*o*_ is the inertia for the module to rotate about its own axis, and *m* and *r* are the module mass and radius, respectively. Therefore, if given the chance, the module will spin around its own axis, rather than travel along the perimeter of another. In future work, we hope to enable gear-like motion via the following approaches: (1) an in-depth study and optimization of SEP and module parameters, (2) synchronized SEP switching in neighboring modules, and (3) addition of friction tape along the module perimeters to make on-axis rotation more energy consuming than translational motion.

### 3.3. Passive Deformation

The passive module deformation is useful both to enable large-scale configurations beyond what manufacturing tolerances would allow with rigid modules, and to permit modules to interact safely with each other modules and their environment. For completeness, we here characterize the deformation modules are capable of.

The components on the flexible PCB are spaced to produce rigid zones, and flexible zones in between the SEPs (Figure 2A). This means that when a static external load is applied to a DONUt module, it deforms by an amount proportional to the load. Beyond guiding the magnetic field, the SEP end caps also function as a mechanical stop which prevents pinching that could permanently deform the PCB. Therefore, when the load is released, the module reverts back to its original shape, exhibiting spring-like behavior. The effective spring constant of a DONUt module was experimentally obtained by applying increment amounts of weight on a flat surface lying on top of a sideways module. The constant is calculated from Hooke's law: *F* = − *k*_*s*_Δ*x*. The term *k*_*s*_ refers to the effective spring constant and Δ*x* is the change in length when a force, *F*, is applied. We found *k*_*s*_ = 28.01 ± 2.85*N*/*m* (Figure 4C).

The looped PCB, of course, does not behave like a perfect spring, and the change in deformation between increment weights decreases slightly with increasing load. This is due to an increasing effect of the rigid zones on the deformation of the module as they are pressed closer to each other at the right- and left-most edges of the module, corresponding to the areas of highest curvature. It should be noted that if the load was dynamic with non-negligible momentum, impact, or vibrations, then the geometric response of the module would be quite different, and it is possible that this effect may be exploited in future work.

### 3.4. Computation

Our choice of controller, ATmega328, coincides with those of the Arduous platforms which are very popular in the do-it-yourself community, again aligning with our philosophy of lowering the barriers to entry for diverse researchers and developers to help increase system robustness. To provide a sufficient number of control pins, each DONUt module has two ATmega328 microprocessors running on their internal 8 MHz RC oscillator, with 2 KB SRAM and 32 KB EEPROM. The first processor controls SEPs 1–7 and three IR transceivers; the second, SEPs 8–12, one IR transceiver, and all SEP communication channels. The two processors communicate via UART. The software for low-level control of all peripheries take up just 2.3% of the SRAM and 6.5% of the EEPROM, leaving the majority of static and dynamic memory for the controllers described in section 5.

### 3.5. Communication

As previously mentioned, we simplify the design by making double use of the SEPs for actuation and module-to-module communication. Restricting the communication range is a commonly used method to avoid bandwidth problems as many asynchronous modules try to communicate (Rubenstein et al., 2014). When two SEPs located on separate modules are in contact, they can communicate locally as follows. The capacitor bank is first charged to maximum capacity. Bits are then transmitted using electromagnetic induction; i.e., the transmitter encodes bits in pulses of current, which are received by the neighboring SEP via induced current in the coil. The receiver then decodes these (weaker) pulses into bits. The current communication protocol is able to send a packet of 4B at a rate of 5 kbps on a single capacitor bank charge.

**Figure 5:**
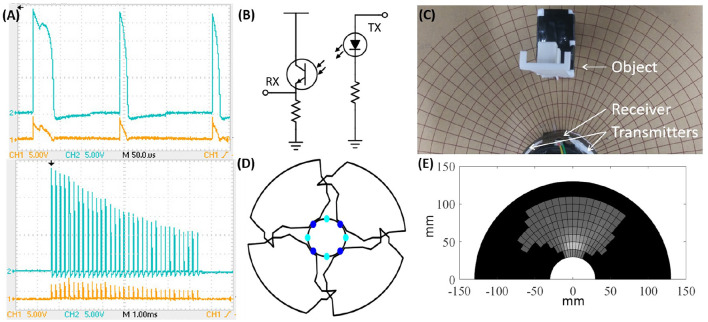
**(A)** Top: Communication packet between SEPs. Received (orange) and transmitted (blue) message of a start bit, “1,” followed by two “0” s. Bottom: Decrease in voltage over the capacitor bank as a package of all “1”s is sent. **(B)** IR transceiver circuit. **(C)** Experimental setup to measure module field of view. **(D)** Top view of the experimentally measured sensor coverage in a module. **(E)** IR intensity map (bright values correspond to close objects and reach a maximum of 498 bits; dark values correspond to 0 and no measured signal).

We developed our own protocol to facilitate communication with bits encoded in pulse length: A “1” is approximately twice the length of a “0” (Figure 5A top). This encoding simplifies synchronization because we can treat any bit like a clock signal, and use a simple schmitt-trigger coupled to a timer input comparator on the processor to decode the package. A transmission is started with a “1,” and bits are sent from least to most significant. The main limitation in baud rate is the time it takes to charge the capacitor bank. Figure 5A bottom shows the decrease in transmission voltage as a (worst case) package of all “1”s is sent, and the capacitor bank discharges.

To test communication reliability, we cycled through a transmission of all possible characters between two SEPs. We found the error rate to be 1 flipped bit per 1,000 bits. This issue may be addressed by adding in one or more parity bits for a slight decrease in throughput.

### 3.6. Sensing

Sensors allow the modules to interact intelligently with their environment. Although we focus on simple IR sensors for gradient tracking and obstacle detection, it is relatively easy to modify the module design to fit different sensors because it only involves a slight re-routing of the PCB.

Currently, each module is equipped with four infrared emitters (OP140A) and four receivers (LTR-301) operating at 935–940 nm (Figure 5B). For full spatial coverage while keeping the number of components small, these eight components are spaced equally around the perimeter of the module, and have a radial emission angle of 40° and a relative sensitivity around 20°, respectively. The outputs from the receivers are multiplexed into the analog to digital converters (ADC) on the processors. To measure the distance to an object for instance, we turn on the relevant emitter and multiplexed channel, and subsequently read the ADC value. We experimentally tested the distance sensors using the setup in Figure 5C. Figure 5D shows a top view of the module coverage pattern and Figure 5E bottom shows the raw values from the sensor. Although every module will have a slightly different coverage dependent on the manual mounting of the sensors, even small objects should be visible before contact. In Ceron et al. (2019b), we discuss how to use this ability for object shape estimation.

### 3.7. Power

A DONUt module can fit up to three single cell 0.15 Ah Li-Po batteries from E-flite, weighing 4.5 g and measuring 45 × 12 × 8 mom each. The module has the ability to measure its own battery level to support more intelligent collective behaviors as further discussed in section 5. The vast majority of energy spent in a module is on actuation and communication. As a rough estimate, a single battery should be able to support *E*_*batt*_/(0.5*CV*^2^) = 6, 000 capacitor bank charges. Given that a single gear-like move requires 11 capacitor bank charges, this corresponds to a full travel length of 12.9 m or 280 module diameters (with no communication). Beyond improved movement, future work will target integration of solar cells to support longer term operation.

### 3.8. Scalability

As argued in the introduction, a focus on individual module robustness supports large scale robot collectives, which in turn enables system-level robustness. Here, we discuss the current state of the modules in terms of cost, fabrication time, and maintenance, and how these may be improved to make large scale DONUt collectives feasible.

#### 3.8.1. Cost

As we have yet to optimize for cost, a single module is priced around 587 USD. The biggest cost stems from the two-layer flexible PCB (468 USD quote from Advanced PCB), the 48 ceramic capacitors (46 USD), the 12 MOSFET drivers (17 USD), and the 12 SEPs (12 USD). The remaining 44 USD stems from components, such as processors, LEDs, resistors, etc.

There are several ways to lower the price of low-volume module fabrication. The cost can be reduced drastically by picking a cheaper PCB manufacturer (the lowest quote was just 90 USD, but had a longer lead time), or by taking advantage of the recent progress in Inkjet printable flexible PCB (Kawahara et al., 2013). The latter reports a drop in price to 10 USD per meter of film, which would leave the cost of the PCB to be negligible compared to the other parts in the module. There are also cheaper alternatives to the current capacitor banks; we could, e.g., use fewer, but larger OSCON capacitors similar to those used for flash in cameras. To give an idea of how the price scales with mass fabrication, the price of the current (non-optimized) component list drops from 119 to 52 USD/module when ordering for 1,000 modules. We aim to produce a second version of these modules with a price point around 50 USD, placing them in a similar range to the cheaper modular robots in literature (Brunete et al., 2017).

#### 3.8.2. Fabrication

One of the key benefits of the DONUt module design, is the reliance on a single PCB which supports imprecise, rapid, and inexpensive manual assembly of both SEPs and wrapped PCB. The largest time sink for the fabrication is component soldering; currently, one PCB takes around 5 h to solder by hand. In the future, we hope to have the majority of the PCB pre-populated at a manufacturing house. To get a rough estimate of how this would trade off cost for lowered assembly time, we requested a quote from Advanced PCB which came to 30 USD/module for an order of 1,000 modules. We expect that this cost can be lowered with a more thorough search of vendors, a longer requested lead time, and the right choice of components. The current capacitor bank, for example, consists of many components in parallel; it would be beneficial to replace these with a few, larger capacitors.

**Figure 6:**
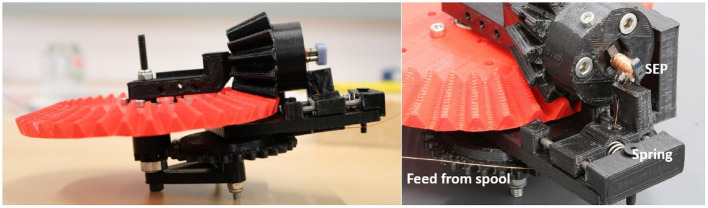
3D printed, manual winding device for rapid production of SEPs. The SEP is mounted into the black piece with a screw; the spring keeps the wire from a spool taught during winding, similar to the mechanism on a sowing machine. A full turn of the red wheel adds roughly 100 turns to the SEP.

If the PCB assembly is outsourced, that leaves the following steps for in-house assembly: (1) SEP manufacturing, (2) attachment of SEPs and batteries, and (3) flexing the PCB into a loop. Of these three, only the first two take any considerable amount of time. The process is as follows. First, the magnet is glued to the steel end caps with super glue; then the assembly is inserted into our winding rig shown in Figure 6. The gears in the rig are dimensioned such that a single turn of the red wheel by hand adds 100 turns to the coil. This entire process, including PCB mounting, takes at most 4 min per SEP, i.e., 48 min per module.

#### 3.8.3. Maintenance

The maintenance requirements of modular robots stem from mechanical wear, the ability of a user to operate (start, stop, and program) all modules with a global command, the module battery life time, and the reliability of individual components. We address each factor in sequence. (1) DONUt modules have no internally moving mechanical parts that can wear with use, and have no loose wires or connectors that may break over time which tend to be one of the bigger problems in small electro-mechanical devices. (2) In future versions, we may explore better parallel operation, enabling user control through a single IR source similar to past platforms (Rubenstein et al., 2014). (3) In the future we may optimize the maximum possible travel distance per module through integration of solar panels on the PCB. Although this type of power harvesting will be slow, it fits this particular style of robots well: only perimeter modules in large collectives are able to move which causes a spiraling migration pattern where the majority of modules at any one point in time remain stationary (further discussed in section 5). (4) Although more thorough tests are needed, we tested 50 moves in a row without any component faults.

## 4. Simulation Environment

**Figure 7:**
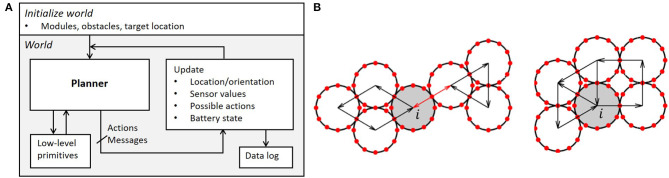
**(A)** DONUt simulation framework. The user specifies the high-level planner, which makes use of low-level primitives. When actions and messages are computed, the framework automatically reevaluates relevant variables, feeds these back to the controller, and logs the state of the world for later debugging. **(B)** Checking if a module (in gray) can move. Left: *i* satisfies props. 1 and 2, but violates 3. Right: *i* satisfies props. 1 and 3, but violates 2, as described in section 4.1.1.

We have developed an open-source simulation platform in Matlab® to support general access to development and testing of control schemes for the DONUts modular self-reconfigurable robot (https://github.com/njw68/DONUts_Simulation). The framework permits programmers to easily test large numbers of modules operating in varying degrees of clutter, and perform structured analysis of system resilience to signal noise and component failures. The simulation incorporates gear-like motion (Figure 3D), connections, sensing range, and message passing. Module compliance, friction, and inter-module forces are not integrated at present. The software is written such that the user can focus on implementation of high-level control schemes, while lower-level primitives like those needed to identify obstacles, connections, and viable motions are abstracted away. An architecture overview is shown in Figure 7.

A programmer can experiment with path planning in cluttered environments with their choice of the number of modules and the amount and complexity of the clutter. The simulation framework may be easily modified to support other task settings and distributed algorithms as well, similar to how we used it in Ceron et al. (2019b). Upon initialization, the programmer may specify the number of modules, the target location, and either pre-determined or randomly generated obstacles with a user-specified size. The software can generate either a rectangular or a random configuration of interconnected modules; it can also run a random initial configuration, where each of the aforementioned variables is randomly generated.

### 4.1. Module Primitives

Next, we introduce several low-level behaviors to support operation of the DONUts.

#### 4.1.1. Motion Restrictions

To determine whether module *i* can physically move, we make three successive checks related to the following properties:

1. *i* is connected to at least one other module, such that at least one *c*_*ij*_ exists, where *c*_*ij*_ is the set of connection positions on *i* between *i* and *j*. *j* refers to the set of modules that are connected to module *i* and *c*_*ij*_ ∈ [1, …, 12].
2. Connections to other modules are contained within 180°, i.e., the module has five consecutive free connections.
3. Movement will not disrupt global connectivity of the modules.

The first property relates to the fact that modules cannot move on their own; the second to the fact that they need physical clearance to move; the third check ensures a cohesive collective (Figure 7B). The latter is done by checking for loops in the connectivity graph; i.e., we pass a message to all neighboring modules to see if it can loop back to the origin without passing the same edge twice. After verifying these properties, we compute the possible movements [clockwise (*CW*)/counterclockwise (*CCW*)/both] taking into account the presence of other modules and obstacles in the environment.

#### 4.1.2. Motion

To physically move a module, it must pass a message to the neighbor which it is rotating about to prepare the next connection (i.e., switch on the correct magnet with the correct polarity). The attraction of the two successive magnets, alongside the repulsion from the previous connection point will propel the module forward. The geometric movement of each module is a function of the center of the module about which they are moving (Figure 3B). We can compute the center position (*x, y*) of a moving module by Equation (2):

(2) $$\begin{array}{l} \left[\begin{array}{c} x_{i}\\ y_{i} \end{array}\right]=\left[\begin{array}{c} x_{j}\\ y_{j} \end{array}\right]+2R\left[\begin{array}{c} cos\left(\theta_{j}+\frac{2\pi }{12}\left(c_{ji}+u\right)\right)\\ sin\left(\theta_{j}+\frac{2\pi }{12}\left(c_{ji}+u\right)\right) \end{array}\right] \end{array}$$

The terms *i* and *j* refer to two adjacent modules; module *i* moves about the perimeter of stationary module *j*. *R* is the module radius, θ_*j*_ is the orientation of *j* with respect to the world reference frame, and *c*_*ji*_ is the magnet position of the connection between *j* and *i* on *j*, where *c*_*ji*_ ∈ [1, …, 12]. The term *u* is the control input for *i* which determines whether *i* will move *CW* or *CCW* about *j*'s reference frame, *u* ∈ [−1, 0, 1]. When *u* = −1, *i* moves *CCW* about *j*; when *u* = 1, *i* moves *CW* about *j*; and when *u* = 0, *i* remains static at its current location.

To keep track of modules and their orientations, we allocate specific IDs to every magnet on the perimeter, and map these to relative IDs as they rotate. An array stores the position of the magnet ID with respect to the inertial frame of reference. Initially, all modules have magnets mapped one to one, such that magnet 1 is at position 1 (*c*_1_ = 1), magnet 2 is at position 2 (*c*_2_ = 2), etc. When a module moves *CCW* about another, the moving module's magnet positions are updated by −1, such that *c*_*k*_ = *c*_*k*_ − 1, *k* ∈ {1, …, 12}. Similarly, *CW* movement results in updates by +1. A check ensures proper rollover when surpassing 1 and 12. The software updates all magnet positions, *c*_*k*_, through the module's control input, *u*:

(3) $$\begin{array}{l} c_{k}\left(t+1\right)=\left(c_{k}\left(t\right)+u\right) \end{array}$$

After the movement has occurred, the module may find itself near new neighbors. To determine the presence of such neighboring modules, a module will briefly activate all connection points, transmit its ID, and await an acknowledge message. In general this sequence needs to be performed only by a module after movement. However, it is possible that occasional checks by all modules to verify their connectivity will improve system robustness.

## 5. Module Coordination

Translating algorithms developed in simulation to real hardware often requires considerable effort. However, such simplified simulations may still be used to quickly iterate the overall coordination methodology as well as to illuminate non-intuitive pitfalls related to the hardware design. In this section, we discuss important findings related to robust coordination of many DONUt modules for gradient tracking, introduced by the hardware-specific constraints. Gradient tracking is a robust and potentially scalable basic behavior necessary for navigation in coordinate-free environments. This behavior may support applications such as identifying the source of chemical spills or simply navigating toward a source of light to harvest power. We introduce controllers for gradient tracking in clutter-free and cluttered environments, using the available sensors described in section 3 and building on the simulation framework and the low level primitives introduced in section 4.

Specifically, we introduce two types of controllers toward robust collective behavior: an A^*^ search-based controller and a more naive, iterative controller. We discuss implementation details, and compare these in terms of complexity and optimality with respect to the number of module moves which directly impacts energy efficiency and maintenance. To produce a benchmark for “optimal behavior,” we also introduce an Oracle planner with complete knowledge of the world. Beyond control methodologies, we discover and discuss a type of connection topology that generally impedes progress along the gradient, and discuss how to avoid this with the naive controller.

Intuitively, sophisticated controllers should not be necessary for gradient tracking as every agent in the collective can simply navigate according to the local gradient. Here, however, we target controllers that advance the entire collective efficiently toward the gradient source. Note that, because (1) we enforce a globally connected collective, (2) modules cannot move on their own, and (3) only perimeter modules are capable of moving, this is not a simple problem. Were we, for example, to perform a naive graph-search across all possible moves of a state in which ten modules are configured in two adjoining rows, this state would have twenty children for a single module move. In other words, the search space quickly becomes intractably large.

**Figure 8:**
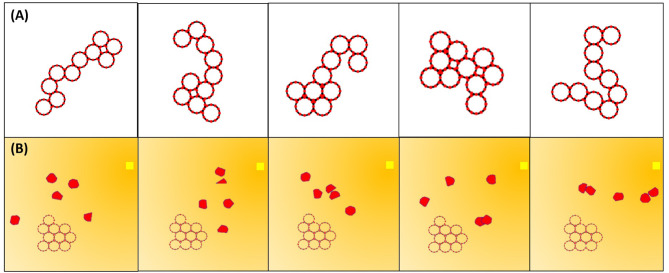
Five examples of the test scenarios for controller evaluation. **(A)** Randomly generated initial configurations (*TS2*). **(B)** Five randomly generated obstacles with randomly generated positions in the path to the goal (*TS3*).

To evaluate our controllers, we use different subsets of the following three scenarios:

- *Test Scenario 1 (TS*1*):* 10 modules starting in a cluster 10 module diameters (20*R*) from the goal in a clutter-free environment.
- *Test Scenario 2 (TS*2*):* 10 modules starting from 10 random configurations 20*R* from the goal in a clutter-free environment (Figure 8A).
- *Test Scenario 3 (TS*3*):* 10 modules starting in a cluster 20*R* from the goal in an environment with five randomly generated and randomly placed obstacles (Figure 8B).

Note that, unless otherwise noted, we abort runs which exceed ~7,000 states; furthermore, we limit the scope to sequential module movement.

### 5.1. Oracle Path Planning

To provide a baseline against which our centralized controllers can be compared, we implement an Oracle planner that computes an optimal path in terms of module moves to a global light source, given complete knowledge of its environment. This planner is based on A^*^ graph-search, where the nodes in the graph correspond to the state of the robot (i.e., the location of all DONUt modules) and the edges correspond to module moves. The cost of a node, *cost*_*state*_, is calculated as the number of moves it takes to get to that state. To search the state space efficiently, we compute an admissible search heuristic, *h*, expressed in module moves, and prioritize nodes with lower total cost: *cost*_*total*_ = *cost*_*state*_ + *h*. The combination of graph-search and an admissible heuristic allows us to prune the large search space and return globally optimal results (Russell and Norvig, 2016). To ensure that the modules cluster around the light source, we complete the search when the distance from the collective center of mass (COM) to the goal is within two module radii, 2*R*.

We examined two heuristics in terms of search space efficiency. The first heuristic, *h*_0_, is based on the intuition that it is beneficial for the collective to quickly align their orientation with the highest gradient. To do this we make *h*_0_ a function of the euclidean distance between the module with the highest measured light intensity (corresponding to the lowest distance to the goal, *D*_*min*_). We compute the number of moves it would take one module to travel this distance and multiply by the total number of modules in the collective, *N*, i.e., *h*_0_ = *ND*_*min*_. The second heuristic, *h*_1_, is more generally based on the intuition that all modules need to move toward the goal. We make *h*_1_ dependent on the euclidean distance between the collective's COM and the goal (*h*_1_ = *ND*_*COM*_). Because modules actually have to travel around the perimeter of other modules to reach the goal, the straight line distance is an underestimate of the true distance and results in an admissible heuristic.

**Figure 9:**
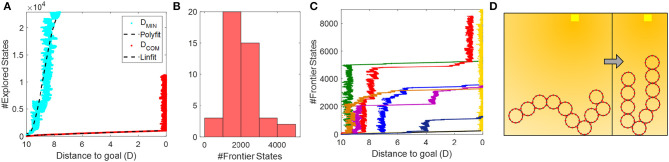
**(A)** Performance of Oracle planner in *TS1* with heuristics *h*_0_ = *ND*_*MIN*_ and *h*_1_ = *ND*_*COM*_. **(B)** Histogram showing the number of states in the graph-search frontier at convergence using *h*_1_, with 55 random initial conditions. **(C)** Examples of the runs shown in **(B)**. **(D)** Example condition that leads to slow search behavior.

We found that *h*_1_ far out-competes *h*_0_ in terms of search space efficiency. An example of what happens is shown in Figure 9A; the number of expanded states in the tree grows exponentially with *h*_0_, and closer to linear with *h*_1_. The intuition behind *h*_0_'s performance is that once a module has been moved as close as possible to the goal within a configuration, all other moves have an equal cost. That is, when a new module moves closer to the goal, but not enough to surpass the current closest module, *D*_*min*_ is the same as the scenario when that module moves away from the goal. In contrast, *h*_1_ ensures that until the collective reaches the goal, we favor states that directly impact the progress of the entire collective. Once the COM gets within 2*R* of the goal, the frontier grows rapidly simply because the heuristic no longer supports closer clustering.

To reason about how well the *h*_1_-heuristic worked, we calculated the effective branching factor, b^*^ in *TS1*. Briefly explained the effective branching factor denotes how many branches every node would have on average if the solution was recast as a breadth first search (*n* = *b*^0^ + *b*^1^ + *b*^2^ + .…*b*^*a*^, where *n* is the number of states, *b* is the number of branches, and *a* is the depth of the search tree). A b^*^ close to 1 indicates that we almost always guess the optimal move and therefore keep the search tree from branching excessively. We found that *h*_1_ is a very efficient guess, with an average b^*^ = 1.034±0.0054, confirming that moving the module that advances the collective COM as much as possible toward the goal is preferred. Note, that this result does not necessarily translate to cluttered environments or take into account the fact that extra connections between modules may improve their redundancy in case of failures.

Interestingly, we found that the search efficiency was heavily dependent on the initial configuration of the collective. To examine this phenomenon more, we ran 55 iterations of *TS2* and plotted the maximum number of states reached in the frontier before convergence. The results are shown in Figure 9B. Forty-seven out of 55 trials converged within the allotted number of expanded states. The fastest searches converged after evaluating about 500 states, but the majority required 2–4 times as many evaluated states. Figures 9C,D illustrates why this occurred. We see that the number of states in the search frontier grows linearly over most of the path, but exhibits periods of exponential growth. These periods occur when the search reaches a state where the collective forms a single chain with the center point of the chain is closest to the goal. In this state, moving either of the two end-modules forward is the fastest predicted way to reach the goal, eventually leading the search to a state in which the collective forms a U-shaped chain. Once this state is found, the search must explore all other higher cost states before it again finds a move that will bring the COM closer than when it was in the U-shape. We designed the following on-board controllers with this risk in mind, to support faster convergence.

### 5.2. A^*^ Search-Based Controller

In a realistic scenario, the modules will not have access to the state of the world and must plan according to what they know; i.e., their connection topology, the measured light intensity, and nearby obstacles. We first implement an A* search-based controller for the modules similar to the Oracle planner with two exceptions. First, instead of planning toward the actual goal, we choose an intermediate goal location which corresponds to the module with the highest measured light intensity, i.e., the module closest to the goal. The modules plan their path to the temporary goal, execute this path, recalculate the new temporary goal, and re-plan. Second, based on the discussion of the effective branching factor, we calculate a new admissible heuristic based on the distance to the intermediate goal from the collective's COM, *h*_2_ = *CND*_*COM*_, where *C* is a constant scaling factor. Effectively, this means that the collective moves in stages (each locally optimal): first communicating and identifying which module is closest to the goal, then planning how to bring their COM to that location before reevaluating which module is now closer. They repeat this process until the collective COM is within 2*R* from the light source.

**Figure 10:**
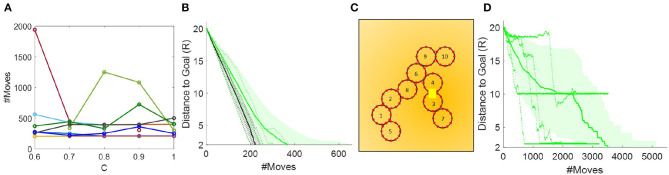
**(A)** Parametric sweep of C in *TS2*. **(B)** Comparison of 10 paths generated by the Oracle (black) and 50 paths generated by the A* search-based controller (green) in *TS2*. The solid lines denote mean, shaded regions the standard deviation, and dotted lines are five actual runs chosen at random. **(C)** Example of live lock near the goal in the A* search-based controller. **(D)** Paths generated by the A* search-based controller in *TS3*.

To find the optimal value of *C*, we did a parametric sweep from *C* = 0.1 − 1.0. We did this sweep with a square initial configuration (*TS1*), and found that *C* ≤ 0.5 performed very poorly, rarely making progress beyond 2*R* toward the goal in the allotted number of states. Conversely, *C* > 0.5 yielded results that were too similar to draw a conclusion. We therefore ran an additional sweep from 0.6 to 1, using 10 configurations in *TS2*. The results are shown in Figure 10A. We found that coefficients at the extremes (0.6 and 1) rarely converged in the allotted number of expanded states. The best results were with *C* = 0.7, which converged in all cases and reached a collective COM within 4*R* and 2*R* from the goal more quickly than when using other values for *C*. Note that with *C* = 0.7, the heuristic is still admissible.

Based on these simulations, we further make the observation that the collective may enter live lock, i.e., an infinitely repeated movement pattern, before reaching the goal depending on the collective's angle to the gradient. An example of what causes this is shown in Figure 10C; modules 10 and 5 oscillate back and forth leaving the collective within the temporary goal, but not the global goal. Because the collective has no memory from previous planning iterations these movement patterns will execute forever. To overcome this, we added a check to assess how much progress the collective has made within one planning iteration. If the collective converge on the temporary goal after moving just one module, no multi-iteration progress occurs. In this case, we move a random module (excluding the last moved module) 1 step. By adding a degree of randomness, we avoid local minima like these, and ensure that the collective will eventually converge at the goal.

In Figure 10B, we next compare the performance of the A^*^ search-based controller to the Oracle in *TS2*. As would be expected, the A^*^ search-based controller is less than optimal. The performance especially degrades as the collective approaches the goal, because at this point the number of moves it takes to reconfigure the collective's COM to the temporary goal dominates the difference of which module is closer to the goal.

We further make the observation that the controller often generates chain-like configurations, where every module on average has only two neighbors. These are problematic because a single module failure can split the chain in two disrupting global performance. The heuristic-based control approach permits a simple way to deal with this issue: we simply add a penalty for a loosely connected graph. Note that this effectively makes the heuristic inadmissible and results in (locally) sub-optimal, but (globally) more robust plans. We used a coefficient α to change the severity of the calculated penalty, *P*, where *x* is the number of connections a module possesses:

(4) $$\begin{array}{l} p=\left\{ \begin{array}{c} x_{i}>2,0\\ x_{i}=2,1\\ x_{i}=1,2 \end{array}\right. \end{array}$$

(5) $$\begin{array}{l} P=\overset{N}{\underset{i=1}{\sum }}p\left(x_{i}\right)\alpha \end{array}$$

**Figure 11:**
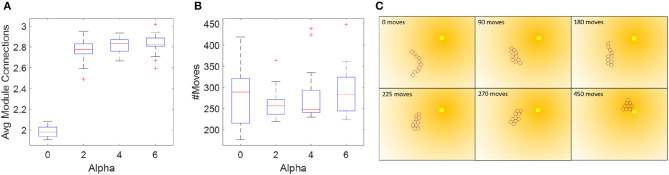
Enforcing a penalty for sparsely connected modules improves the redundancy of the system and is independent of the time to reach the goal. **(A,B)** Number of module moves and connections as a function of clustering penalty α. Each box plot shows the median, standard deviation, outliers, and the 25th and 75th percentiles based on 20 runs in *TS2*. **(C)** Example path generated with an α = 6.

In other words, we add a penalty of 2α for modules that are configured in a chain and only have two neighbors, and a penalty of α for modules that are at the end of a chain. The new cost per node comes to: *cost*_*total*_ = *cost*_*state*_ + *h*_2_ + *P*. We ran this simulation in *TS2* using α = [0 2 4 6], and compared both the total number of moves needed for the collective's COM to reach the goal within 4*R* and the average number of connections in each step along the way (Figures 11A,B). Again, we see that the initial configuration has a big impact on performance, and that, as expected, with increasing penalty, the modules stay more clustered. The choice of α relates both to the desired redundancy and the number of modules in the collective. For example, with ten modules configured in a double-row the average number of connections per module corresponds to 3.4. We see that the graph levels out at α = 2, i.e., at 2.8 connections per module which is reasonable given that some modules have to deviate from the double row for the collective to move. This experiment is a repeated measures, correlated samples test, thus we perform a one-way ANOVA for correlated samples and find that α has a statistically significant effect on the average number of module connections [*F*_(3, 57)_ = 663, *p* < 0.0001]. Conversely, α does not have a statistically significant effect on the number of module moves [*F*_(3, 57)_ = 1.32, *p* = 0.28]. The average number of moves between the αs vary by ~30. To explain this, we examine the simulations at α = 0 and α = [2 4 6]. We find that while with a value of *C* = 0.7 and α = 0 is an admissible heuristic for local optimization, it results in chain-like configurations, which are more susceptible to temporary live lock. These instances of temporary live lock require modules to move sub-optimally to break out of temporary live lock and resume regular planning. This causes an increase in the number of modules moves to reach the goal. In the cases of α = [2 4], we observe that clustered configurations lead to sub-optimal local planning, but more robust global planning, thus fewer temporary live lock instances occur, and the average number of module moves is less than with α = 0. An example of the path taken given α = 6 is shown in Figure 11C. This brief study indicates that adding a clustering penalty is a viable and simple way to ensure higher collective redundancy.

Finally, we tested the A* search-based controller in cluttered environments (*TS3*). The results shown in Figure 10D indicate live lock near obstacles. By studying the actual runs closely, we find that this happens because the number of modules which can move randomly is severely limited by either their connection topology (chain-like configuration) or their proximity to obstacles. We may deal with this by adding either a higher degree of randomness or memory between planner iterations. The former comes at the cost of planner efficiency and without guarantees that live lock can be avoided in all situations. The latter is complicated because the collective may enter configurations that appear similar to previous ones, but at geographically different locations. Modules may compute their trajectory to overcome this problem, however, this would require perfect dead reckoning skills which is not practically feasible with the hardware.

We can further discuss the ability of this planner to operate on the actual hardware processor, i.e., the 2 KB of RAM in the ATmega328P (Ceron et al., 2019a). The state space of the planner grows somewhere between linear and exponential with the number of modules, depending on (1) the optimality of the heuristic and (2) the configuration of the collective, i.e., the number of modules that are capable of movement. Every node in the search tree contains the collective's connection topology and a reference to the parent and child nodes. For 10 modules in a perfect cluster this would correspond to 19 connections and 1 parent node, i.e., a memory footprint of 20B. In this state, 8 modules are capable of moving *CW*, *CCW*, or staying, therefore the node has 24 children; i.e., just two levels in, the search would take up 500 B of memory. Alternatively, we could trade off memory for computation by storing only the move and recomputing the configuration for every explored node. In this case we spend 20 B on the first node, and 1 B per node moving forward. With a good heuristic, the memory would then grow close to linear as in a depth-first search. To improve memory, we could further explore how this search could be distributed to over the two on-board processors (Colbrook and Smythe, 1990). Given the current hardware constraints, however, we are unlikely to be able to support planning for more than a few tens of modules. In the next section, we instead focus on more naive, iterative planners that require less memory and computation altogether and are inspired by what we learned from the graph-based controllers.

### 5.3. Naive, Iterative Controllers

To produce an algorithm that scales well in memory and computation with near-optimal control, we next examine a naive, iterative controller for gradient tracking. In this controller, we simply prioritize moves of modules that are farthest from the light source, hence we name this type farthest-first or “faf-controllers.” As before, we identify the module with the highest light intensity, i.e., the one closest to the light source, and treat it as an intermediate goal. We then identify a movable module with the lowest brightness, and move it toward the intermediate goal along the shortest path around the collective perimeter. This process repeats until all modules are clustered around the goal location. We explore two versions of this controller. In the first, we move the darkest module one step before searching for a new darker module (“faf0”). Intuitively, this approach works well for highly dynamic environments where information quickly becomes stale. In more static environments, or when communication between modules or between modules and the centralized controller is costly, the update rate can be lowered by only re-planning when the moving module reaches its intermediate goal (“faf1”).

**Figure 12:**
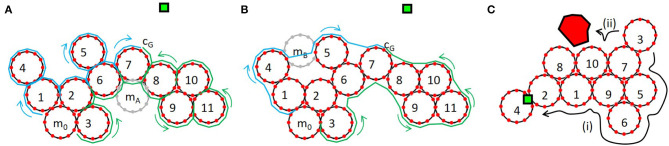
**(A,B)** Example configuration in which module *m*_0_ plans a path, either *CW* (blue) or *CCW* (green), to the location with the highest brightness, *c*_*G*_. **(C)** Illustration of the two versions of the faf1 controller. In faf1_*i*_ *m*_3_ chooses the only viable path toward the goal; in faf1_*ii*_ it follows the shortest path to the goal and stops when it sees an obstacle.

The following list details how this controller works, using the example shown in Figure 12.

1. *Initialize:* We first identify the intermediate goal, i.e., the brightest module, *m*_7_; and the darkest module which is capable of moving, *m*_0_. We also identify which connection on *m*_7_ is closest to the light source, *c*_*G*_.
2. *Generate viable paths and identify external obstacles:* We next decide which direction (*CW* or *CCW*) around the collective will yield the most efficient path. We construct two candidate sequences, *S*_*CW*_ and *S*_*CCW*_, along the perimeter modules from *c*_*G*_ to the closest connection on *m*_0_, *c*_0_. To construct *S*_*CCW*_, we first append *c*_*G*_ (*S*_*CCW*_ = [*m*_7_(*c*_*G*_)]) and then continue appending connections on *m*_7_ in a CCW direction until the neighboring module, *m*_6_, is reached (*S*_*CCW*_ = [*m*_7_(*c*_1_:*c*_*G*_)]). We continue this process until we reach the *m*_0_ (*S*_*CCW*_ = [*m*_1_(*c*1:*c*7)]…*m*_6_(*c*_1_:*c*_4_)*m*_7_(*c*_1_:*c*_*G*_)]). These steps unambiguously define the sequence of connection points that *m*_0_ must go through to reach the goal. In other words, by using connection points rather than module center points, we avoid confusing any connection on the closest module (e.g., *m*_*A*_ in Figure 12A) with the goal location. Every time we encounter a new module, we check for obstacles seen by that module to indicate whether or not the sequence is tenable.
3. *Optimize viable paths and identify geometrically incompatible modules:* The second step overestimates the number of moves needed to reach the goal, because it does not take into account locations like *m*_*B*_ illustrated in Figure 12B, where the module can connect directly between two non-neighboring modules, *m*_4_ and *m*_5_. To prevent this overestimate, we loop through each tenable sequence, projecting *m*_0_ along the sequence generated above, checking for physical proximity of connectors that would reveal new potential neighbors. By iterating forward through the sequence and for each projection checking backwards from the goal we ensure that *m*_0_ always identifies the connection that is closest to the goal. If one exists, we delete all of the intermediate entries and produce new sequences, *CW*′ and *CCW*′. If at any point the *m*_0_ projection overlaps with another module we label the sequence untenable. Note again, that overlap is possible because we are not operating in a discrete occupancy grid. On the real hardware, overlap could also occur because of module deformation.
4. *Move module along shorter path:* Finally, we simply compare the length of the sequences and move the module in the direction of the shortest path.

**Figure 13:**
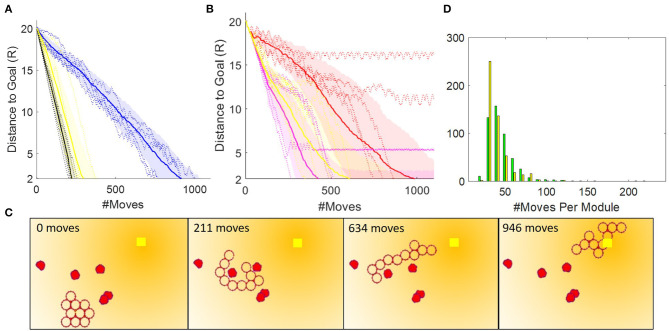
**(A)** Ten paths generated by the Oracle planner, and 50 paths generated each by the faf0 (blue) and faf1 (yellow) controllers in *TS2*. **(B)** Fifty paths generated by the faf1_*i*_ (red), faf1_*ii*_ (magenta), and faf1 (yellow) controllers in *TS3*. The solid lines denote mean, shaded regions the standard deviation, and dotted lines are five actual runs. **(C)** Snapshots of a path generated by the faf1 planner. **(D)** Histogram showing the number of moves per module. To compute this plot, we counted all moves per module from 50 runs in *TS2* with the A* search-based and the faf1 controller. Note that we discounted runs that reached live lock near the goal.

Figure 13A shows the performance of faf0 and faf1 in *TS2*. Because the test is performed in a static, clutter-free environment faf1 outperforms faf0, here by a factor of ~4. The oscillations in faf0 occur when the collective, similar to what we saw with the Oracle planner, finds itself in a U-shaped chain where it greedily moves the darkest module up the gradient at each cycle, effectively making the collective gather at one extreme of the connection topology, then the other, until it finally reaches the global goal. We see that faf1 performs almost as well as the Oracle planner, but that the performance is still dependent on the initial configuration.

For operation in cluttered environments, we explored three variations of faf1, also illustrated in Figure 12C. In faf1_*i*_, if the shortest path which the darkest module must take to the intermediate goal is intercepted by an obstacle, we instead move the module in the opposite direction; if obstacles are detected in both directions, we move another module. In faf1_*ii*_, when an obstacle is encountered we simply move the darkest module as close to the obstacle as possible. In faf1, when an obstacle is encountered, we choose according to faf1_*i*_ and faf1_*ii*_ with 50% likelihood, and, with 20% likelihood move a random movable module one step in a *CW* direction. Figure 13B compares the performance of these three variations in *TS3*. Generally, faf1_*ii*_ outperforms the others, however, it may enter livelock. Similar to our previous observations, we find that this happens in U-shaped chain configurations where the two ends point toward the goal and are near obstacles that hinder further movement. At this point each end module simply moves back and forth along the collective, without making actual progress. faf1_*i*_ does not show issues with live lock, but take nearly twice as long to reach the goal. faf1 overcomes issues with live lock due to stochasticity, at the cost of ~1.5 times more module moves. An example path generated by faf1 is shown in Figure 13C.

Deriving the exact scaling behavior for this planner is complicated due to the motion restrictions discussed in section 4.1.1. In the algorithm, most operations scale constant or linear with the number of modules; however, optimizing the path along the collective, i.e., step number 3 in the description above, approximates polynomial time. Intuitively explained, in the worst-case scenario where the collective is spaced out in a single file line and not in the presence of obstacles, the darkest module has to be projected along every other module to check for short-cuts. This step is an interesting point for future work. Another obvious direction for improving the scalability of this algorithm is to outsource computations. In Ceron et al. (2019b) we, for example, detail how the connection topology can be computed in a distributed manner. To extend the current planners to a completely distributed system, one can imagine combining these algorithms with a consensus-based scheme to identify the modules with highest and lowest brightness.

### 5.4. Discussion

In summary, in the context of gradient tracking in clutter-free environments, our 10-module simulations indicate that both controllers may perform nearly optimal despite the lack of global knowledge. The locally-optimal A* search-based controller performed well in terms of the number of modules moves for clutter-free environments, but additional measures must be taken to overcome potential live lock near obstacles. We also find that even with a good search heuristic, the algorithm scales poorly in terms of memory and will not support more than a few tens of modules if implemented on the two on-board ATmega328 processors. The naive, farthest-first controller performed equally well and had the ability to deal with live lock near obstacles via a small degree of randomness. This controller is simple and may support more scalable behavior.

Through simulations, we further found that both types of controllers generally create chain-like, rather than clustered, configurations. Chain-like configurations are bad for the DONUts, because (1) they severely limit the amount of modules that are capable of moving, (2) simulations show that chains often end up creating U-shaped configurations that impede general progress toward the goal, and (3) they leave the collective at risk of complete failure if just a single module breaks. We showed that encouraging redundant connection topologies in the A* search-based controller was fairly simple; encouraging these in the farthest-first controllers will be an important area of study in future work.

Finally, system energy consumption warrants explicit discussion because it is a major contributor to system autonomy and robustness, affecting the strategy of exploration vs. exploitation as the modules traverse an environment. The DONUt hardware, for example, was designed around SEPs which keep their polarization without continued supply of power, the number of power consuming components was minimized, and the modules were designed as light weight as possible (25.4 g). In Figure 13D, we compare the A* search-based and the faf1 controller in terms of how well they distribute energy consumption among the modules. As we have yet to consider energy spent on communication in our centralized controller, the energy we can estimate is directly correlated with the number of moves a module has to make. The plot shows that the faf1 controller inherently distributes power usage more evenly, whereas a few modules in the A* search-based controller moves many times further than the others. Evening out power consumption will also be an interesting future extension to our work.

## 6. Conclusion

In summary, we have introduced a new planar, modular, self-reconfigurable robot. Although more work is needed before practical large-scale demonstrations are feasible, this initial hardware-software design cycle has contributed several concepts that may translate to other platforms. Most importantly, by basing our design on a single flexible PCB without mechanically moving parts, we were able to achieve simple, fast manufacturing, and support low maintenance in terms of breakage and wear. By creating an open source simulation platform with realistic movement and sensing, we explored two control schemes and non-intuitive challenges that arose because of the module-specific motion constraints. We explicitly focused on enabling a large configuration space to enable operation in dynamic environments, and explored a range of challenges related to collective efficiency, scalability, redundancy, and adaptability. More generally, we showed that enabling scalability and system-level robustness, rely on tightly integrated design decisions that span fabrication, operation, and control with an explicit focus on constituent robustness.

We have several agendas moving ahead. On the hardware side, we will focus on decreasing cost, increasing battery life, and improving motion reliability before pursuing a large-scale collective. So far, we have depended only on the passive compliance for added robustness, however, long term, we hope to investigate novel collective behaviors enabled by the compliance, including their ability to generate macroscopic materials with different density and tensile strength. Similarly, their spring-like properties promises interesting dynamic behaviors which may be leveraged for both communication and motion. Finally, the fact that every single module weighs only 25.4 g also indicates a new set of potential applications beyond those seen with previous platforms. On the control side, we are exploring several avenues. The most near-term is to combine centralized and decentralized algorithms for better scaling properties. Longer term, we hope to better investigate the trade-off between control redundancy and efficiency. A video description of this project can be found in Supplementary Material.

## Data Availability Statement

The datasets generated for this study are available on request to the corresponding author.

## Author Contributions

All authors collaborated on the contents of this article. Hardware design and characterization was lead by SC and LH. Simulation environment by SC and NW. Path planners by NW.

### Conflict of Interest

The authors declare that the research was conducted in the absence of any commercial or financial relationships that could be construed as a potential conflict of interest.
